# The quest for tolerant varieties: the importance of integrating “omics” techniques to phenotyping

**DOI:** 10.3389/fpls.2015.00448

**Published:** 2015-07-09

**Authors:** Michel Zivy, Stefanie Wienkoop, Jenny Renaut, Carla Pinheiro, Estelle Goulas, Sebastien Carpentier

**Affiliations:** ^1^Department Génétique Quantitative et Évolution, Le Moulon INRA, CNRS, AgroParisTech, Plateforme PAPPSO, Université Paris-Sud, Gif-sur-Yvette, France; ^2^Department of Ecogenomics and Systems Biology, University of Vienna, Vienna, Austria; ^3^Department of Environmental Research and Innovation, Luxembourg Institute of Science and Technology, Belvaux, Luxembourg; ^4^Instituto de Tecnologia Química e Biológica, New University of Lisbon, Oeiras, Portugal; ^5^Faculdade de Ciências e Tecnologia, New University of Lisbon, Caparica, Portugal; ^6^Department of Sciences et Technologies, CNRS/Université Lille, Villeneuve d’Ascq, France; ^7^Department of Biosystems, University of Leuven, Leuven, Belgium; ^8^SYBIOMA, University of Leuven, Leuven, Belgium

**Keywords:** proteomics, data integration and computational methods, phenotype, omics-technologies, crop improvement

## Abstract

The primary objective of crop breeding is to improve yield and/or harvest quality while minimizing inputs. Global climate change and the increase in world population are significant challenges for agriculture and call for further improvements to crops and the development of new tools for research. Significant progress has been made in the molecular and genetic analysis of model plants. However, is science generating false expectations? Are ‘omic techniques generating valuable information that can be translated into the field? The exploration of crop biodiversity and the correlation of cellular responses to stress tolerance at the plant level is currently a challenge. This viewpoint reviews concisely the problems one encounters when working on a crop and provides an outline of possible workflows when initiating cellular phenotyping via “-omic” techniques (transcriptomics, proteomics, metabolomics).

## Introduction

The need for higher yields with lower inputs is widely recognized as necessary to meet the challenge of feeding 9 billion people in 2050 (Godfray et al., 2010). Agricultural management is being challenged by erratic climates and the occurrence of extreme stress events that have the potential to destroy crop production in many geographical regions. Stress is complex and involves timing, duration and severity (Blum, 2014). Moreover, plant stress in agriculture is a phenomenon that is correlated with the genotype, environment and management (G × E × M). Breeding toward stress tolerance is limited in many crops and the current commercially grown varieties have mainly been selected for production and excellent post-harvest qualities, with less attention to other features (e.g., drought tolerance, nutrient use efficiency, durable pest and disease resistance, environmental repercussions etc.). The effects of these factors have been/are mitigated by the use of treatments such as irrigation, pesticides and fertilizers. These management processes have reduced the sense of urgency and resulted in the use of existing plant genetic resources in overcoming crop limitations. Consequently, several ancient varieties and landraces or even wild crop relatives containing useful sources of resistance or tolerance are now underutilized. However, the use of pesticides, fertilizer and water must be reduced and agriculture must become more sustainable. Recently, many governments have commenced initiatives to promote plant germplasm collections which increase the range of material that can be explored in search of genotypes less affected by stress^1^^,^^2^. Reliable identification of tolerant varieties and the understanding of their genetic diversity are urgently needed. The knowledge gap is a strong propeller for the generation of biological knowledge (both fundamental and applied) and provides the plant biology community with the opportunity to establish different experimental models (genomics, transcriptomics, proteomics, metabolomics, phenomics) for different crops. Phenotyping is an emerging field that characterizes plant behavior and quantify features, such as growth and yield, in a way that allows linking to genetic control. However, the evaluation of genetic biodiversity is a research bottleneck and there is still a significant gap between the lab and the field. Is science generating false expectations? Are ‘omic techniques, such as proteomics and metabolomics, generating valuable information that can be translated into practice? Is proteomics better than metabolomics, transcriptomics or genomics? Recently, a European network was created to help tackle this issue and develop new workflows to integrate the different ‘omic techniques: COST action FA1306 “The quest for tolerant varieties—Phenotyping at plant and cellular level^3^.” The aim of the Action is the improvement and exchange of scientific knowledge in plant phenotyping through the creation of a network between European interdisciplinary scientists and to use this network to: map valuable gene bank collections and breeding programs in Europe, train breeders and physiologists in screening techniques and data interpretation, get insight into the genetic basis of tolerance, to characterize current biodiversity and rank it according to tolerance levels and to apply the knowledge for agricultural management. The Action started in May 2014 and 28 countries have currently joined. This viewpoint embodies the vision of this COST action and describes concisely the problems one encounters when phenotyping the diversity found in crops. It specifically provides an outline of the problems encountered when initiating “cellular phenotyping” through ‘omics techniques, highlighting proteomics.

## Understanding Gene function

Understanding gene function can be approached via several techniques: genomics, transcriptomics (messenger, structural and regulatory RNA’s), proteomics (proteins and their putative post-translational modifications (PTM) and peptides) and metabo-lomics (primary and secondary metabolites). In prokaryotes, gene finding is essentially a matter of identifying open reading frames. As genomes get larger, it becomes increasingly complicated. Several sophisticated software algorithms have been designed to handle gene prediction in eukaryotic genomes. Despite considerable progress, gene prediction entirely based on DNA analysis is cumbersome and requires support from “functional genomics,” i.e., transcriptomics, proteomics, and metabolomics. Indeed, genomics focuses on the static aspects of genome information. Gene prediction and annotation in a reference variety is the initial step for every crop, but this is not sufficient to get complete insight into the phenotypic plasticity and the agricultural potential of the biodiversity. Functional genomics deals with dynamic aspects, reflecting environmental adaptations and allows the description of gene functions as well as the interactions between gene products that may provide a view of the agricultural potential of a variety/genotype.

## Transcriptomics

Probably the easiest way to study changes on a genome-wide scale is through transcriptomics. The structure of RNA is homogenous and relatively simple and therefore the analysis is the most straightforward when compared to protein and metabolite analyses.

Serial analysis of gene expression (SAGE), developed by Velculescu et al. (1995), is based on the generation of 15 bp tags from a defined position in each transcript, which are then concatenated, cloned into a plasmid vector and ultimately sequenced (Velculescu et al., 1995). Massively parallel signature sequencing is a more advanced technique based on sequencing of tags. It generates 17 bp tags that are sequenced using a fluorescence-based signature sequencing method on microbeads and was applied on multiple model organisms (Brenner et al., 2000; Reinartz et al., 2002). To analyze the abundance of a transcript, one simply calculates the number of times that a certain tag was found. Though no sequence data needs to be identified *a priori*, the tag needs to be identified as belonging to a gene to convey its biological meaning and this step can be difficult in plants with limited genetic resources. DNA sequences are not as well conserved as amino acid sequences and therefore a cross-species identification based on a short tag is problematic. Matsumura et al. (2003) developed superSAGE in rice, which utilizes longer tags (26 bp). However, the generation of longer tags still resulted in the SAGE-approach for un-sequenced non-model crops challenging, as illustrated for banana (Coemans et al., 2005; Carpentier et al., 2008a). At the same time microarrays, which were significantly cheaper and was a more high-throughput technology, were also developed for transcriptomic studies. Microarrays use known probes that will hybridize with the labeled sample and based on the intensity of these dyes, transcript levels are estimated. This however, implies that sequence information exists before generation of the microarray and this is a serious limitation when applied to non-model crops. The limited sequence availability in non-models can be overcome by the use of microarrays of closely related species or by the generation of a species-specific microarray based on known expressed sequence tag (EST) data for instance, but these analyses will be less informative (Davey et al., 2009; Pariset et al., 2009). Microarray analysis is hampered by a high background noise due to cross-hybridization as well as saturation of signals. Microarrays therefore have a limited sensitivity and dynamic range. Furthermore, microarrays are closed platforms as unknown transcripts cannot be detected. An alternative for the standard gene expression microarray is the tiling microarray. These are high-density arrays composed of oligonucleotide probes that span the entire genome of an organism (Yazaki et al., 2007). Whole-genome tiling arrays may provide part of the solution toward the detection of new gene transcripts in a sequenced organism but are more expensive and still suffer from the general drawbacks of a microarray approach (Valdés et al., 2013). With the availability of next generation sequencing (NGS) technologies, the possibility to directly sequence mRNA at relatively reduced costs became available. This technique, termed RNA-seq, has clear advantages over the other transcriptomics methods: a higher sensitivity and dynamic range can be achieved (Wang et al., 2009) and no previous sequence knowledge is *per se* required. Reads can be mapped to a known reference genome or *de novo* assembled. *De novo* assembly of reads into contigs increases the use of this technique for crops whose genome has not been sequenced, as was demonstrated for wheat, agave and horse gram (Bhardwaj et al., 2013; Gross et al., 2013; Oono et al., 2013). However, a reference genome is highly recommended to assure the correct assembly of the reads and to deal with paralogs and allelic variants. Moreover, most read-mapping software has been written to analyze diploid genomes (Langmead and Salzberg, 2012) and is unsuited for polyploid organisms (Page et al., 2013a). Read mapping is a fundamental part of next-generation genomic research but is complicated by genome duplication in many plants. When a reference genome is already available, RNA-seq can provide additional information necessary to identify previously unknown gene coding sequences. Categorizing DNA sequence reads into their respective genomes enables current methods to analyze polyploid genomes as if they were diploid. Page et al. (2013a,b) developed software for SNP detection in cotton, which is an allotetraploid. Using SNP-tolerant mapping, the software uses the SNPs between genomes to categorize reads according to their respective genomes. Furthermore RNA-seq data can also be used to improve existing annotations both in identifying actual intron-exon structures as well as in identifying different splice variants as was shown in maize (Kakumanu et al., 2012) or identifying homeologs. Genome-wide quantification of homeolog expression ratios was technically hindered because of the high homology between homeologous gene pairs. Additionally, in contrast to the high background noise caused by cross-hybridization in microarrays, most RNA-seq reads can be unambiguously mapped to a region of the reference genome. This makes RNA-seq in combination with reference genomes an excellent tool to differentiate between isoforms of a gene family, which are a widespread phenomenon in complex crop genomes. On the other hand, the alignment of short sequence reads that are shared between several loci and therefore align to several locations on the genome is still complicated. One solution is to assign these reads proportionally to the number of unique splice reads at these loci (Mortazavi et al., 2008). Moreover, aside from being relatively unbiased toward previous sequence knowledge, RNA-seq is also more sensitive. This sensitivity comes at a price. To detect rare transcripts, coverage and therefore sequencing depth is required, which increases the sequencing cost. Lastly, the dynamic range of RNA-seq is also substantially higher, at about five orders of magnitude compared to several hundred-fold for microarrays (Wang et al., 2009; Zhao et al., 2014).

With the introduction of NGS, RNA-seq appears to be the transcriptomic tool for the future, especially in crops. At the moment, the costs associated with RNA-seq prevent large scale analysis of many varieties in different conditions and multiple biological replicates. However, as the NGS technique keeps evolving, costs are likely to drop and may no longer be a limiting factor in the future. As more and more genomes are sequenced, alignments to reference genomes should become standard practice, which will also significantly reduce the analysis time required for *de novo* assembly.

## Proteomics

In contrast to genomics and transcriptomics, proteomics is often regarded as a slow and cumbersome art. The discovery of soft ionization techniques for mass spectrometry (MS) by Nobel Prize winners Fenn and Tanaka, the coupling of MS to liquid chromatography and the genomic and computational advances, have made the high throughput large scale analysis of proteins feasible (Karas and Hillenkamp, 1988; Fenn et al., 1989; Henzel et al., 1993; McCormack et al., 1997). Thus, after a significant lag phase, high throughput proteomics has become an important research tool for model organisms and is currently finding its way to crop species.

Two approaches are generally distinguished in the field of proteome analysis: a protein based approach (in general, referred to as gel based) and a peptide based approach (in general referred to as gel free or shotgun). In the gel based approach, proteins are separated and quantified via gel electrophoresis. The proteins of interest are then picked from the gel, digested and the resulting peptides identified via MS by comparing experimental *versus* theoretical masses present in various databases. This technique has the advantage that protein separation and analysis via (two-dimensional) electrophoresis prior to MS analysis ensures physical connectivity between the peptides and the protein and significantly reduces complexity (Carpentier et al., 2008b). Currently, it is still the most widely used approach in crop proteomics. Unfortunately the technique has some major drawbacks, i.e., it has a very poor performance when analyzing hydrophobic and basic proteins and can be quite limited with respect to throughput.

In the gel free approach, protein digestion precedes the separation and quantification of peptides. Gel free differential proteomics provides a broader coverage of the proteome and also enables the identification of membrane proteins. However, a major disadvantage of this approach lies in the disconnection between the protein and its peptides (Carpentier and America, 2014). In general, most approaches use a bottom-up strategy where proteins are first digested with a proteolytic enzyme. Yates and colleagues were one of the early pioneers to explore the use of liquid chromatography coupled to electrospray ionization tandem mass spectrometry (LC/MS/MS) and realize the potential of automated high throughput proteomics (McCormack et al., 1997; Ducret et al., 1998; Link et al., 1999). However, proteolytic digests of a higher eukaryotic proteomes, like crops, exceed the analytical capacity of most MS. During recent years, MS have been developed with high mass accuracies, resolving power, sensitivity, scan speed, reproducibility and lower detection limits (Domon and Aebersold, 2006; Mann and Kelleher, 2008). For example, the use of Fourier transform ion cyclotron resonance based spectrometry (Yang and Yen, 2002), hybrid Linear Trap Quadrupole-Orbitrap devices (Makarov et al., 2006; Olsen et al., 2009), high energy C-trap dissociation (Olsen et al., 2007), parallel reaction monitoring (Peterson et al., 2012), the coupling of a quadrupole mass filter to an Orbitrap analyser (Michalski et al., 2011; Kelstrup et al., 2012), Ultra Performance Liquid Chromatography (UPLC) combined with moist static energy (MSE; Plumb et al., 2006), combining quadrupole, Orbitrap and ion trap mass analysis (Lebedev et al., 2014), and hybrid quadrupole time-of-flight MS (Andrews et al., 2011), have all contributed to improvements in proteomic experiments, and in particular toward better peptide identifications and quantification. Despite the development of new MS, a protein sample from a crop species is still challenging to analyze and contains several thousand proteins. This might lead to both identification and quantification problems, especially in the case of crops with complex polyploid genomes and large protein families. Peptides shared between several proteins do not contribute to the conclusive identification of a particular protein. This is the so-called protein inference problem (Nesvizhskii and Aebersold, 2005). Unique peptides need to be measured and identified for final protein identification and quantification. So a gel free approach is only applicable for crops, in practice, once a reference genome is available or when substantial EST libraries become available (Vertommen et al., 2011b). Typically, a gel free analysis starts with an MS survey scan where peptide precursor masses are measured, followed by an MS/MS scan for fragmentation of the selected precursor ion. This is called data-dependent acquisition (DDA). The serial nature of the MS and MS/MS cycles and the complexity of the proteome in crops remain a challenge. There is a bias toward the more abundant peptides and no MS scan can be obtained while fragmentation is being performed in the MS/MS scan in most current MS. Moreover, co-eluting peaks can lead to chimeric spectra, reduced reproducibility and loss of information about less abundant peptides. To increase the chance of identifying specific tryptic peptides, it is important to ensure a good peptide separation and to keep the mixture of co-ionizing peptides as simple as possible even in fast modern MS. Vertommen et al. (2011a) proposed a workflow for banana samples where this was achieved by using a two-dimensional RP-RP chromatography system coupled to a high accuracy MS. To identify the peptides in a gel free approach for a crop, it is important to build a species specific in-house database and to search this database in consecutive steps: a non-error tolerant manner, subsequently an error tolerant and finally *de novo* sequencing. This *de novo* approach is a crucial step, since it allows the identification of variety specific peptides via a homology search of sequences instead of a search based on m/z values (Vertommen et al., 2011a).

To identify and quantify peptides in a rapid, consistent, reproducible, accurate and sensitive way, data independent acquisition (DIA) protocols have been developed for label-free shotgun proteomics as an alternative to DDA (Plumb et al., 2006). To increase the identification rate of label free DIA experiments for samples from the apple variety Braeburn, a new workflow was developed by Buts et al. (2014) where a DDA database was constructed and linked to the DIA data. A ten-fold increase in peptides was identified from a single DIA run and proteins correlated to the storage quality of apples were found.

While in sequenced crops, two-dimensional electrophoresis (2DE) may currently no longer be the tool of choice for high-throughput differential proteomics, it is still very effective in identifying and quantifying protein species as a result of genetic variations, alternative splicing and/or PTM. As an example, by using combined 2DE and 2D DIGE with *de novo* MS/MS sequencing, Carpentier et al. (2011) were able to identify inter-and intra-cultivar protein polymorphisms in banana correlated to drought tolerance. Using an 2D-DIGE LC MS/MS approach Vanhove et al. (2015) were able to characterize the complex protein family of HSP70s and identify an osmotic stress specific isoform. Likewise, the molecular mechanisms for rapid metabolic responses to stress remain largely unknown and to fill this gap, the role of PTMs needs to be investigated. As an example, in response to cold stress, which involves quick adjustments to the photosynthetic machinery, many cold-acclimation-related proteins are putatively regulated by PTMs, as has been recently highlighted in pea by using 2D-DIGE analysis (Grimaud et al., 2013).

## Metabolomics

In the ‘omics field, metabolomics generates large datasets for the identification and quantification of small molecules. Usually, the approach is undertaken for the high throughput detection of secondary (flavonoids, sugar-phosphates, phytohormones, phytoalexins, etc.) and primary metabolites (sugars, organic-and amino acids, etc.). Complementary MS-based LC and GC approaches are required to adequately profile this metabolic diversity (Scherling et al., 2010). Although it is possible to gather tens of thousands of metabolic variables, their accurate identification remains the major bottleneck in metabolomics studies to date. Like for proteins, due to the large dynamic range and the difference in physico-chemical properties, detecting the entire “metabolome” is not possible. In contrast to proteomics, for metabolomics analyses, functional identification is not dependent on the availability of genome sequence information. However, the availability of purified standard substances and/or spectral libraries/databases is necessary. Of the various MS methods, two profiling strategies can be typically distinguished: a targeted and a non-targeted approach (Wienkoop et al., 2008, 2010). The non-targeted technique allows for the quantitative evaluation of unknown, yet unidentified, variables. Nevertheless, metabolomics databases and the availability of MS-derived metabolite fragment information are increasing. Furthermore, novel strategies for pathway and structural assignments of untargeted high-throughput metabolomics data are being developed. For example, an algorithm termed mzGroupAnalyzer was developed to investigate metabolite transformations caused by biochemical or chemical modifications, and the approach led to the identification of novel molecule structures (Doerfler et al., 2014). Specifically, it resulted in the detection of 15 unknown, putative cold and light stress regulated metabolites of the flavonoid-pathway in *Arabidopsis thaliana*. Metabolomics is also playing an increased role in stress marker detection and contributing to improved stress tolerance in crops, as previously reviewed (Hirayama and Shinozaki, 2010; Weckwerth, 2011; Martinez-Gomez et al., 2012; Rasmussen et al., 2012). Metabolomics has also contributed to the detection of putative marker(s) induced by the pathogen *Rhizoctonia solani* in potatoes (Aliferis and Jabaji, 2012) and soybean (Aliferis et al., 2014) as well as bacterial blight-resistance in rice (Wu et al., 2012).

## QTL Analysis

Most traits of interest for crop breeding are controlled by multiple quantitative trait loci (QTL), and the major objective of using ‘omics in this context is the characterization of these QTLs. Analyzing the genetic variations of transcripts, proteins and metabolites at a large-scale allows the search for causal relationships between molecular and phenotypic variations with a global approach and without *a priori* knowledge. The study of natural genetic variations is not only interesting for breeding purposes, but also to decipher the biological processes involved in the genotype to phenotype relationship. Indeed, it is not uncommon that loss of function mutations have no clear phenotype, and the analysis of small disturbances caused by QTLs may allow a better understanding of metabolic pathways and regulatory networks involved in the variations of the phenotypic trait (Sulpice et al., 2010).

Genotyping is the information on which all breeding programs are based to map the QTLs, to measure kinship between genotypes or populations, to analyze changes in allele frequency during selection, etc. In recent years, considerable advances have been made in genotyping and hundreds of thousands of SNP markers (single nucleotide polymorphism) are available today, at relatively low cost for crops whose genome has been sequenced. This information is subsequently used to identify candidate genes or proteins. Overall, the strategy consists of mapping QTLs of transcript expression (eQTLs) or abundance proteins (PQLs or pQTLs), and looking for co-localization between them and QTLs of the traits of interest. The underlying idea is that QTL/(eQTL or PQL) co-localizations may be due to a single polymorphism that causes quantitative or qualitative (amino acid polymorphism) variation of the transcript and/or protein, that in turn would be the cause of the variation of the trait of interest. When these co-locations also co-localize with the gene encoding the transcript or protein, then the causal polymorphism is likely located within the gene, including the promoter region (*cis*-QTLs). This gives breeders the opportunity to select the favorable allele itself, which is far more efficient than using QTL flanking markers.

When QTL/(eQTL or PQL) co-localizations are found outside the region of the gene (*trans*-QTLs), the QTL can be any sequence that influences the protein or transcript abundance. In this case, it is not precisely identified; nevertheless the co-localization indicates a possible involvement of the gene/protein in the genetic variation of the phenotypic trait. This information is interesting both for the breeder and for understanding the mechanisms involved in the variation of the trait. As co-localizations can also be found due to chance, candidate genes or proteins are selected according to *a priori* knowledge on gene function or regulation that support the involvement of the gene in the studied trait. After validation, the information brought by these co-localizations can be used in breeding programs by selecting alleles that influence the abundance of the transcript or protein, or by transgenesis to over- or under-express these genes.

As can be deduced from the discussions above, each ‘omics approach offers advantages and drawbacks. Transcripts are direct gene products, and thus the link between genomic information and expression data is quite simple. On the other hand, the link between transcript abundance and the phenotype is loose, because of the multiple steps from transcripts to protein, from protein abundance to activity and metabolite concentrations, and from metabolites to cellular, physiological and plant phenotypes. Quite a number of eQTL analyses have been performed in plants, including in crops. For example, Li et al. (2013b) mapped a total of 30,774 eQTLs for 22,242 genes by RNA sequencing a maize population of intermated recombinant lines. Thirty-seven percent were *cis*-eQTLs, while the other 63% were *trans*-eQTLs. The latter were often grouped in hotspots. In many of these hotspots, the effect of alleles from the same parent affected gene expression in the same direction. The genes controlled by hotspots were often enriched in a particular functional category. The last two observations suggested that hotspots contain upstream regulators controlling cellular processes. *Cis*- and *trans*-eQTLs have been observed in various proportions according to the species and the study, and hotspots showing some kind of functional specialization are also often observed (Kliebenstein, 2009). Several eQTL studies have been performed with the aim of identifying candidate genes. For example, Li et al. (2013a) identified in 2013 74 loci highly associated with maize oil concentrations or composition by genome-wide association study (GWAS). The expression of all 41 genes at these loci was controlled by *cis*-eQTLs and for 32 it was correlated to the targeted or related traits. Sequencing five of them in a collection of genotypes allowed the identification of polymorphisms in their UTR or promoter regions, which was likely the cause of the variation of their expression and for the variation of kernel oil content and composition. Most eQTL studies have been carried out by analyzing segregating populations, but when the objective was to target a particular QTL, near isogenic lines (NILs) were also used (Bolon et al., 2010; Kugler et al., 2013). Bulk segregant analysis, where different genotypes are mixed according to the genotype in the QTL region, were also used (Chen et al., 2011).

Metabolite QTLs (mQTLs) have also been mapped both in crops and in model organisms (Kliebenstein, 2009). Metabolites are products of biochemical reactions catalyzed by proteins (enzymes), and as such they are closer to the phenotype than transcripts. On the other hand, the relation to genomic sequences is tenuous, because metabolite amounts may depend on many other biochemical reactions than those directly involved in their synthesis or degradation: many enzymes can be directly or indirectly responsible for the variation of a single metabolite. Many of the mQTL studies or analyses of the natural genetic variability of metabolites were performed with the goal of linking metabolite variations to other biochemical, physiological or phenotypic traits. Kerwin et al. (2011) compared *Arabidopsis* mQTLs to eQTLs of genes and showed a single peak of expression per day. The results, combined with the analysis of mutants, allowed them to conclude that variations in glucosinolate content can influence the internal circadian clock. Sulpice et al. (2013) analyzed the relationships between metabolism and biomass in a panel of 97 *Arabidopsis* accessions and observed that correlation-based networks were very specific to growth conditions. Desnoues et al. (2014) analyzed the correlations between sugars and enzymatic activities in peach fruits in a progeny of 106 genotypes. Interestingly the variations in sugar content was only poorly explained by the variations of enzymatic capacities of the enzymes directly involved in their synthesis or degradation, suggesting that their variations could be related to changes in other components of sugar metabolism.

In several mQTL studies, parallel mapping of eQTL or analysis of gene expression were performed to identify candidate genes. For example, Brotman et al. (2011) identified a gene encoding a putative fumarase in the region of a fumarate mQTL in *Arabidopsis*. They showed that the fumarate content was greatly reduced in mutants and that the expression of the candidate gene was 16 times more highly expressed in the parent that showed the highest level of fumarate. These results suggest that there is a causal relationship between the genetic variation of the expression of this gene at this locus and the natural variation in fumarate content. A similar strategy was followed by Zorrilla-Fontanesi et al. (2012) to identify a candidate gene for the production of mesifurane by the strawberry fruit.

Some proteins are in-between transcripts and metabolites in the genome-to-phenotype relationship, since they are effectors of biological processes and other proteins are real end products influencing the phenotype directly. In proteomics studies, the link between genes and proteins can be ambiguous in particular because of peptides shared between members of gene families. On the other hand, as proteins are the results of transcription, transcript turnover and translatability, post-translational events and protein turnover, their amount represents the integration of many processes that lead to the cellular and *in fine* plant phenotype. Consequently, correlations between protein and transcripts amounts are relatively low (Gygi et al., 1999; Carpentier et al., 2008a; Schwanhausser et al., 2011) which supports the necessity to analyze proteome variations. Wilhelm et al. (2014) have calculated the protein/mRNA ratio for many protein and transcripts in a certain tissues and claim that knowing the translation rate constant, it becomes possible to predict protein abundances with good accuracy from the measured mRNA abundance. This is because the translation rate constant is a dominant factor in determining protein abundance (Wilhelm et al., 2014). Some further confirmatory experiments will need to be undertaken to confirm whether this hypothesis is adequately supported.

The first PQLs were mapped in 1994 (Damerval et al., 1994) and the candidate protein strategy was developed in 1999 (de Vienne et al., 1999). It allowed the identification of the ZmASR1 candidate protein for tolerance to drought, whose effect on yield was then confirmed by over-expression (Virlouvet et al., 2011). The ASR protein was also found to be relevant in other species, e.g., tomato, grape, lily and banana (Maskin et al., 2001; Cakir et al., 2003; Wang et al., 2005; Henry et al., 2011). Segregating populations were also used to map seed and leaf protein PQLs in pea and wheat (Amiour et al., 2003; Bourgeois et al., 2011; Legrand et al., 2013). More often, proteomics has been used to search for candidate proteins in relation to a particular QTL, by using NILs (Hajduch et al., 2007; Torabi et al., 2009; Bernardo et al., 2012; Gunnaiah et al., 2012; Lesage et al., 2012; Li et al., 2014). The analysis of bulked genotypes grouped according to their phenotype can also be useful. It has helped in identifying candidate genes in potato as a complement to a study based on association genetics (Fischer et al., 2013).

To our knowledge, no study on the genetic diversity of PTMs has been performed, although it is well known that protein phosphorylation can play an important role in plant response to biotic and abiotic stresses (Bonhomme et al., 2012; Rampitsch and Bykova, 2012). Although the quantification of PTMs is more difficult than the analysis of protein abundance, and that even identifying a correlation between a modifying enzyme and its target is challenging, it is likely that “candidate PTMs” would be of great interest for QTL characterizations. The analysis of large numbers of genotypes is necessary for PQL mapping or genome wise genetics association studies. The constant progression of the performances of MS-based quantitative proteomics will allow those types of analysis to be performed in the near future, to achieve in plants what has already begun in yeast (Foss et al., 2007; Picotti et al., 2013; Skelly et al., 2013) and in humans (Wu et al., 2013).

## Data Integration

Prior to the development of high throughput methods for extensive metabolome, proteome, transcriptome, genome and recently phenome research, scientists dreamed about the integration of different datasets to gain a deeper insight. Data integration, as a tool to enable in-depth insights into processes, can be performed at two levels. On the one hand, one can integrate homogeneous data derived from measurements on the same entity made in the same experimental set-up. On the other hand, meta-analysis can be applied to integrate heterogeneous data derived from different experiments (Dupae et al., 2014). The latter approach poses a challenge as the integration of data performed using different standards and methods are difficult. Thus, we will focus on the integration of homogenous data derived from the same experimental set-up that has been used to get a clearer view on plant (dys)function. Over the past few years, questions on how to unravel the existing links between the metabolome, proteome, transcriptome, genome and the phenome from a particular entity have been discussed. As well as how this knowledge can provide better insights in mechanisms like abiotic stress tolerance or biotic stress resistance. Statistical multivariate methods have been refined to uncover those links and are now used to integrate two or even more datasets based on predefined models of one-by-one dataset relationships (Le Cao et al., 2011; Gunther et al., 2014; Tenenhaus et al., 2014). This not only makes it possible to integrate multiple ‘omic levels, but even enables scientists to “supervise” and direct such analyses toward meaningful relationships between the ‘omics datasets (Gunther et al., 2014).

A major advantage in integrating metabolomics with proteomics is gaining spatial and temporal information on the end products upon environmental constrains. Since specific protein isoforms can target a specific protein to particular tissues and/or compartment, the use of integrative subcellular fractionation and localization strategies will allow the detection of dynamic distributions within the cell. The temporal plasticity of metabolism constitutes various phases of adjustment. In order to capture the interaction between the metabolome and the proteome, it is necessary to investigate the system along a period of time. Correlative network- as well as Granger-Causality (time-series correlation) analyses have been demonstrated to be effective tools in obtaining information on pathway interplay and reprogramming cues (Doerfler et al., 2014). Linking ‘omics data with such mathematical approaches facilitates the interpretation of time dependent chronological processes and the identification of variables being controlled by time-lagged values of other variables.

The next step is to link the transcriptome/proteome/metabolome to plant performance (phenome), i.e., linking cellular phenotyping to plant phenotyping. The detailed characterisation (physiological, cytological, biochemical, gene expression, protein and metabolite profiles) of different plant species represent a real and difficult challenge. The necessary resources and or knowledge are most likely not present in one research group or country. This is where a European network such as the COST project can make the difference. An integration of these data into regulation/interaction networks will represent new and important advances for understanding plant responses. A multivariate analysis approach becomes of interest because the algorithms are designed to explain as much of the variability at the plant level as well as the variability at the cellular level. This method is especially useful to assess polygenic traits such as drought tolerance, because one can specifically look for cellular and plant level variations that explain the variation in genotypes and treatments used. In this way candidate genes related to important tolerance mechanisms at the plant level can be retained for further analysis, a procedure called feature selection. Retained candidate genes can further be related to the kyoto encyclopedia of genes and genomes (KEGG), gene ontology (GO), protein domain, protein family, protein function, and more categorical databases, as a form of heterogeneous data integration (Gomez-Cabrero et al., 2014). Identification of key genes, pathways, regulation networks of metabolism and stress responses, in association with physiological stress-related data, represent crucial information that have to be integrated and presented in web databases. This way the physiological context of the genes of interest can be compared to the literature to confirm the obtained results.

## ‘Omics and Breeding: From Lab to Field

The genetic gain obtained via breeding programs supports the yield gain of new crop varieties. In this way it is relevant to increase the genetic base available for selection, i.e., the genetic diversity, and also to characterize the molecular and phenotypic consequences of such diversity. Germplasm characterization through ‘omics allows one to perform the molecular characterization of genotypes, providing a list of candidate genes/gene products that are highly valuable for breeding and/or engineering stress-tolerant crops with novel traits (Mir et al., 2012; Pinheiro et al., 2014). The characterization of genetic diversity in germplasm collections is typically performed through DNA based markers. However, the use of non-DNA markers (as transcripts, proteins and metabolites) has the advantage to provide information on the molecular interactions and networks operating in a given genotype; these have the potential to help evaluate the potential of the different genotypes. As indicated above, each technique focuses on a subset of the biological interaction network and each technique has its strong and weak points. At present, blind high throughput proteomics and metabolomics studies are often regarded as being descriptive. This might be due to the fact that not many of the putative markers that have been proposed have been evaluated and transferred to the productive sector. The reason might be that high throughput crop proteomics and metabolomics are still emerging ‘omic approaches with limited resources and high associated costs. Due to cost, setup, training, requirement for labs /greenhouses etc, most studies are limited in size and number of biological replicates. In many cases these numbers are far too low. Currently the number of studies in crops exploring diversity via different ‘omic techniques is limited. Germplasm screening and/or discrimination between genotypes of *Phaseolus vulgaris* (Mensack et al., 2010), *Oryza sativa* (Heuberger et al., 2010), *Miscanthus* (Straub et al., 2013), and *Hordeum vulgare* (Heuberger et al., 2014) are some examples. Technologies keep evolving and become more powerful and cheaper. Proteomics and metabolomics are powerful tools when combined with a good experimental design and when the candidates are validated under realistic conditions. It is essential to identify potential traits under lab conditions that are responsible for superior yields under realistic field conditions in different environments.

The current breeder’s toolbox makes use of genetic molecular markers, QTLs, gene expression and biochemistry and phenotypic (morphological and agronomic) data for target traits. The use of these tools is dependent on the breeding objective (phenology, yield, yield components, quality, disease, adaptation) and on the crop. While available tools can differ from crop to crop, the crop growing habit (cool vs warm season crops) and the agricultural system also needs to be considered. For each crop, and for each climatic zone, major constraint(s) need to be identified and goals established. The strategy of “one size fits all” is not well suitable, and regional and local goals need to be addressed (climate, soil, social). Another important issue is the availability and the costs of each technology. Langridge and Fleury (2011) present a summary of ‘omic resources available for some crops.

**FIGURE 1:**
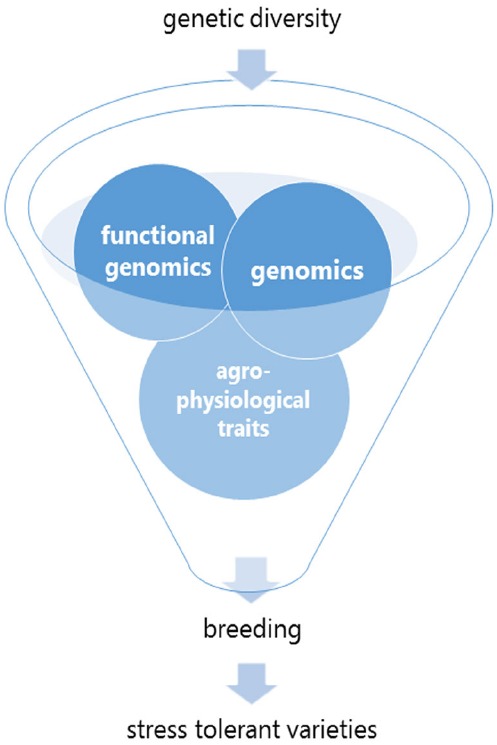
**Genetic diversity and breeding tools.** After having defined the breeding objective(s), the ideal toolbox makes use of genetic molecular markers (QTLs), data from functional genomics (transcriptomics, proteomics and metabolomics) and integrates it to the phenotypic (morphological and agronomic) data for target traits.

In the 21st century, the research and agricultural community face a challenge to deliver new stress tolerant and productive crops. Does science and ‘omic techniques generate false expectations? No, ‘omics technology is powerful and promising when combined with relevant experimental design and subsequently validated in a realistic environment. The use of the ‘omics generated knowledge is a promising tool in the breeders tool box (Figure 1). A dialog and consultation between breeders, farmers, researchers from natural and social sciences and politicians is required. This is where the COST project “The quest for tolerant varieties—Phenotyping at plant and cellular level” needs to contribute. Through a strong dialog between stakeholders and their cooperative activity it will be possible to deliver better agricultural products that utilize less inputs, have lower environmental costs and provide higher levels of social well-being.

### Conflict of Interest Statement

The authors declare that the research was conducted in the absence of any commercial or financial relationships that could be construed as a potential conflict of interest.

## Footnotes

1 http://www.croptrust.org/

2 http://www.cgiar.org/

3 http://costfa1306.eu/
